# Reactive Oxygen Species Regulate T Cell Immune Response in the Tumor Microenvironment

**DOI:** 10.1155/2016/1580967

**Published:** 2016-07-28

**Authors:** Xinfeng Chen, Mengjia Song, Bin Zhang, Yi Zhang

**Affiliations:** ^1^Biotherapy Center, The First Affiliated Hospital of Zhengzhou University, Zhengzhou, Henan 450052, China; ^2^Department of Oncology, The First Affiliated Hospital of Zhengzhou University, Zhengzhou, Henan 450052, China; ^3^Department of Hematology/Oncology, School of Medicine, Northwestern University, Chicago, IL 60201, USA; ^4^School of Life Sciences, Zhengzhou University, Zhengzhou, Henan 450052, China; ^5^Engineering Key Laboratory for Cell Therapy of Henan Province, Zhengzhou, Henan 450052, China

## Abstract

Reactive oxygen species (ROS) produced by cellular metabolism play an important role as signaling messengers in immune system. ROS elevated in the tumor microenvironment are associated with tumor-induced immunosuppression. T cell-based therapy has been recently approved to be effective for cancer treatment. However, T cells often become dysfunctional after reaching the tumor site. It has been reported that ROS participate extensively in T cells activation, apoptosis, and hyporesponsiveness. The sensitivity of T cells to ROS varies among different subsets. ROS can be regulated by cytokines, amino acid metabolism, and enzymatic activity. Immunosuppressive cells accumulate in the tumor microenvironment and induce apoptosis and functional suppression of T cells by producing ROS. Thus, modulating the level of ROS may be important to prolong survival of T cells and enhance their antitumor function. Combining T cell-based therapy with antioxidant treatment such as administration of ROS scavenger should be considered as a promising strategy in cancer treatment, aiming to improve antitumor T cells immunity.

## 1. Introduction

Reactive oxygen species (ROS) are small short-live oxygen-containing molecules that are chemically highly reactive. Of more than 20 types of ROS, superoxide anions (O_2_^•−^), hydrogen peroxide (H_2_O_2_), and hydroxyl radicals are the most important subtypes contributing to cell damage and even death [1]. ROS are generated mainly by following mechanisms: xanthine oxidase-dependent, respiratory chain and NADPH oxidase- (NOX-) dependent pathways. Mitochondrium is a major place to produce intracellular ROS, and complexes I and III of electron transport systems are main sources of mitochondrial O_2_^•−^ [2]. In addition, there are also other exogenous sources of ROS, including ultraviolet and gamma radiation, air pollutants, and chemicals [3–5]. Superoxide anion generated initially in cell is converted rapidly into H_2_O_2_ freely crossing cell membranes, which can be further converted into hydroxyl radicals in the presence of Fe^2+^ or Cu^2+^ [6]. Compared to other ROS, H_2_O_2_ has a longer half-life (about 1 ms in an aqueous solution) than other ROS (<1 *μ*s) and functions as an important oxidant in microenvironment [7, 8]. Moreover, H_2_O_2_ reacts with thiols at a physiological concentration and forms disulfide bond [9]. Thus, H_2_O_2_ can act as a second messenger because of the following: (i) it has relative long half-life, (ii) it is uncharged, (iii) it can cross membranes, (iv) it is relatively specific (thiols), and the modifications (disulfide bonds) are reversible [10]. H_2_O_2_ has been reported to participate in many processes, such as cell growth, stem cell renewal, tumorigenesis, cell death, cell senescence, cell migration, oxygen sensing, angiogenesis, circadian rhythm maintenance, myofibroblasts differentiation, and immune responses [7, 11–16].

ROS elevated in almost all cancers act as a double-edged sword during tumor development [17]. For example, ROS-mediated DNA damage triggers malignant transformation of cells and promotes cancer initiation. ROS levels are also associated with cancer cell stemness [18]. It has been demonstrated that immunosuppressive tumor microenvironment facilitates tumor invasion, metastasis, and resistance [19]. ROS are likely immunosuppressive participants in tumor progression [20]. Indeed, ROS production greatly contributes to inhibitory activities of tumor-induced-immunosuppressive cells [21, 22]. Therefore, ROS are not only mediators of oxidative stress, but also players of immune regulation during tumor development. ROS-mediated signaling can be additionally regulated via altering local concentrations (e.g., using antioxidants) [23]. ROS are essential particularly at low levels for a wide range of innate immune functions, including antiviral, antibacterial, and antitumor responses [24]. This review will mainly discuss the production of ROS in the tumor microenvironment and the impact on antitumor T cell immune response.

## 2. ROS Generation in the Tumor Microenvironment

**Figure 1:**
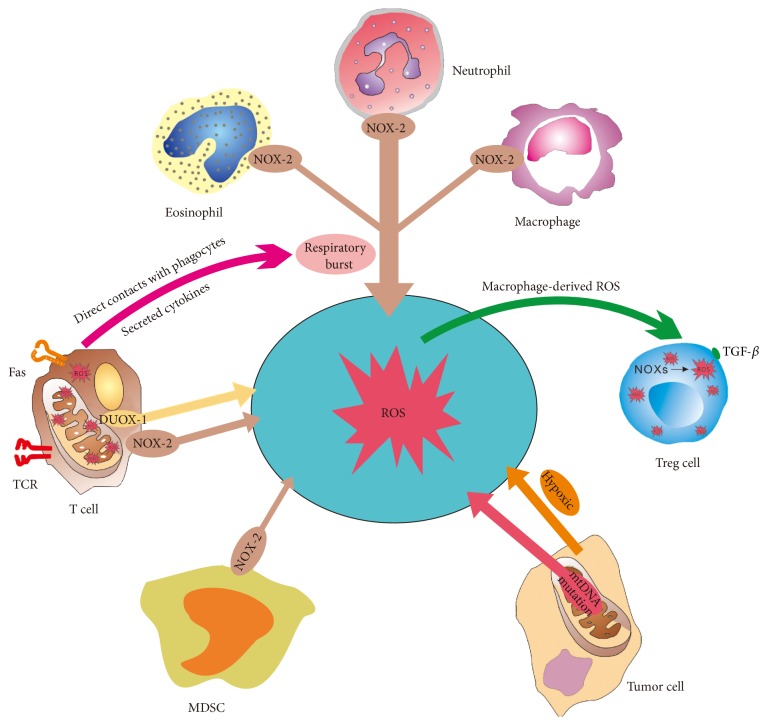
ROS produced in the tumor microenvironment. FasL ligation and TCR signaling in T cells could induce the production of ROS via NOX-2, DUOX-1, and mitochondria. Activated phagocytes (neutrophils, eosinophils, and mononuclear phagocytes) can produce large amounts of ROS by the NOX-2 during respiratory burst. Activated T cells can also induce respiratory burst by direct contacts with phagocytes or cytokines. TGF-*β* activates NOXs of Tregs, which trigger the production of ROS. Moreover, macrophage-derived ROS can induce Tregs accumulation in the tumor microenvironment. Mutations of mitochondrial DNA (mtDNA) in tumor cells result in a deficiency in respiratory complex I activity and contribute to the overproduction of ROS. MDSCs also produce amounts of ROS in the tumor microenvironment.

As shown in Figure 1, ROS produced by cancer cells and tumor-infiltrating leukocytes, including myeloid-derived suppressor cells (MDSCs), tumor-associated macrophages (TAMs), and regulatory T cells (Tregs), can suppress the immune responses.

It has been revealed that MDSCs, as one of the major immunosuppressive subsets, play a pivotal role in promoting tumor progression and contribute to suppressive tumor microenvironment by producing ROS [25, 26]. Furthermore, it has been reported that administration of ROS inhibitors completely abrogated the suppressive effect of MDSCs on T cells [27]. ROS reduce T cell immune responses via inhibiting recognition between T cell receptor (TCR) and MHC-peptide complex, while adding ROS inhibitors such as catalase into the MDSCs/T cells coculture system could impair suppressive effects of MDSCs on T cell proliferation [28]. MDSCs isolated from mice lacking NOX-2 showed little or no ROS production and also failed to suppress the proliferation and IFN-*γ* production of T cells [29, 30]. It has been reported that MDSCs inhibited T cell activation by depleting cystine and cysteine [31], which is closely correlated with ROS production. Indeed, cystine and cysteine are essential for synthesizing the glutathione (GSH) that eliminates ROS production. In addition, scavenging of H_2_O_2_ with catalase induces differentiation of immature myeloid into macrophages in tumor-bearing mice, suggesting that ROS also play an important role in maintaining the undifferentiated state of MDSC [32, 33]. However, low level of ROS could activate T cells and anti-CD3 induced phosphorylation of extracellular signal-regulated ERK pathway required H_2_O_2_ generation [34].

TAMs are considered as critical links between inflammation and cancer development [35, 36]. ROS produced by macrophages have been reported to have immunosuppressive properties and could also be functional for induction of Tregs [37]. The ROS producing capacity by different subtypes of macrophages is discrepant. M2-type macrophages induced by M-CSF and IL-10 have a higher ROS producing capacity [38]. In contrast, CD137, a costimulatory immune checkpoint molecule, could reduce typical macrophage characteristics such as phagocytosis, oxidative burst, and CD14 expression, which could induce the differentiation of monocytes to dendritic cells (DC) and DC maturation and reduce ROS generation [39]. ROS produced by macrophages were higher than those by DCs [40] while CD137L-activated microglia induce apoptosis of oligodendrocytes dependent on ROS [41].

Apart from MDSCs and macrophages, T lymphocytes are another main source of ROS. Indeed, peripheral blood T lymphocytes from cancer patients showed an increased ROS production compared to those from healthy subjects [42]. The process of TCR activation is accompanied by ROS production, and tumor-infiltrating lymphocytes could be dysfunctional due to the ROS accumulated in the tumor microenvironment. Intracellular ROS level in T cells is tightly regulated through NOX-2, dual-substrate oxidase 1 (DUOX-1), mitochondria, and the expression of a variety of antioxidant systems, including superoxide dismutase, peroxiredoxins, and glutaredoxins coupled to metabolic status of T cells [43–45]. The major sources of ROS production in T cells are lipid metabolism, mitochondria, and NOXs [44, 46]. Mitochondria generate low amounts of ROS (superoxides) in a controlled and stimulation-dependent fashion, thereby less likely to have a direct influence on tumor cells or other surrounding cells. DUOX-1 activation generates H_2_O_2_ that acts in a positive feedback loop to enhance and stain further TCR signaling [45]. However, high amounts of extracellular ROS produced by an oxidative burst from macrophages or in a pathophysiological condition induce the disability of T cells [38, 47]. Interestingly, low amounts of ROS can stimulate T cell activation/proliferation [48, 49]. Tregs are key immunosuppressive cells increased in cancer patients. TGF-*β* secreted by Tregs activates the NOXs to produce ROS. Low level of ROS has been also shown to induce the immunoregulatory enzyme, indoleamine 2,3-dioxygenase, and enhance the function of Tregs [50]. Tregs exhibit reduced sensitivity to ROS-induced cell death, while the level of ROS determines the function of Tregs. Indeed, Tregs isolated from neutrophil cytosolic factor 1 (Ncf1) deficiency mice with a lower level of ROS were hyporeactive compared to those from wild type mice [50].

Other inflammatory cells such as neutrophils, eosinophils, and mononuclear phagocytes could produce ROS in the tumor microenvironment as well [51], thereby contributing to tumor growth and antitumor immune response.

Besides immune cells in the tumor microenvironment, tumor cells could also generate excessive ROS [42], which may be encoded from mutations of electron transport chain (ETC) mitochondria-related genes as well as the mitochondrial DNA damage. For example, a loss of p53 causes depletion of mitochondrial DNA and altered homeostasis of mitochondrial ROS [52]. ROS generated by mitochondria contribute to the initiation of nuclear of mitochondrial DNA mutations that promote neoplastic transformation [53]. ROS in cancer cells can be also driven by increased metabolism, oncogene activity, and abnormal expression of NOXs and play a doubled-edged sword role in cancer progression. The dual roles of ROS depend on their concentration [54]. On one hand, ROS could facilitate carcinogenesis and cancer progression at mild-to-moderate elevated levels. Metabolic synergy or metabolic coupling between glycolytic stromal cells (Warburg effect) and oxidative cancer cells occur in cancer and promote tumor growth, while ROS are key mediators of the stromal Warburg effect [55]. On the other hand, excessive ROS would damage cancer cells dramatically and even lead to cell death [54, 56]. Tumor cells can express increased levels of antioxidant proteins to detoxify ROS [57]. Nuclear factor erythroid 2-related factor 2 (Nrf2) is a pivotal transcription preventing oxidative stress, but aberrant activation of Nrf2 often occurs in various human cancers. Silencing Nrf2 inhibited proliferation of glioma cells via AMP-activated protein kinase- (AMPK-) activated mammalian target of rapamycin (mTOR) [58]. In contrast, capsaicin mediates bladder cancer cell death through increasing ROS production [59]. Hypoxia-inducible factor 1*α* (HIF-1*α*) can induce ROS production by acting on complexes I, II, and III of mitochondria ETC [60, 61]. Both HIF-1*α* and nuclear factor-*κ*B (NF-*κ*B) could induce the expression of MMPs to promote ROS production by regulating COX-2 in tumor cells [62]. Indeed, activities of MMP-2 and MMP-9 in tumor tissues were correlated with superoxide radicals generation rate [63]. Taken together, considering dual roles of ROS, the strategies of decreasing or increasing the level of ROS in cancer cells warrant cautious consideration for cancer treatment.

## 3. Regulators of ROS Production

During the process of ROS production, the level of ROS is usually regulated by many factors in the tumor microenvironment. First, there are several checkpoints restricting ROS production by the NOXs following activation of receptors by ligands such as insulin, platelet-derived growth factor, transforming growth factor, nerve growth factor, fibroblast growth factor, tumor necrosis factor-*α*, and epidermal growth factor [64–67]. Second, when tumor diameters reach about 200 *μ*m, tumor tissues become hypoxic, representing a negative prognostic indicator [68, 69]. Hypoxia induces ROS production through regulating transcription Nrf2 that reduces ROS accumulation [70]. MMPs have been identified as important regulators of the activity of mitochondrial respiratory chain and intracellular ROS production [71]. Third, ROS generation was associated with cell metabolism and glucose metabolism and mitochondrial respiratory would increase ROS production [49, 72]. In addition, Calnexin expression is required for cellular NOX4 protein expression and ROS formation [73], which may regulate cell apoptosis induced by endoplasmic reticulum stress or by inositol starvation [74, 75]. Camalexin induced T-leukemia Jurkat cell apoptosis by increasing ROS concentration and activation of caspase-8 and caspase-9 [76]. Several chemotherapeutic agents, such as Chelerythrine (protein kinase C inhibitor) and Quinones, also induced tumor cells apoptosis through increasing ROS [77, 78].

Level of ROS is dynamic and regulated by antioxidant system in the body. Antioxidant mechanisms, either enzymatic (catalases, dismutases, and peroxidases) or nonenzymatic (vitamins A, C, and E and GSH), are critical to protect cells against ROS-induced damage [1]. ROS-mediated signaling can be opposed by specific antioxidants. For example, GSH, a major intracellular redox molecule that protects cells from oxidative stress [79], is essential for optimal T cell proliferation and activation, and it is synthesized by cysteine [80]. Inactivation of the extracellular superoxide dismutase (SOD) leads to accumulation of ROS in the tumor microenvironment [81]. Manganese superoxide dismutase (MnSOD) is a major antioxidative enzyme, neutralizing O_2_^•−^ released by electron chain as a by-product of respiration. Silencing MnSOD results in increasing intracellular oxidative stress, while increasing MnSOD exerts an antitumor effect both* in vitro* and* in vivo* [82].

## 4. ROS Affect T Cell Activation

**Figure 2:**
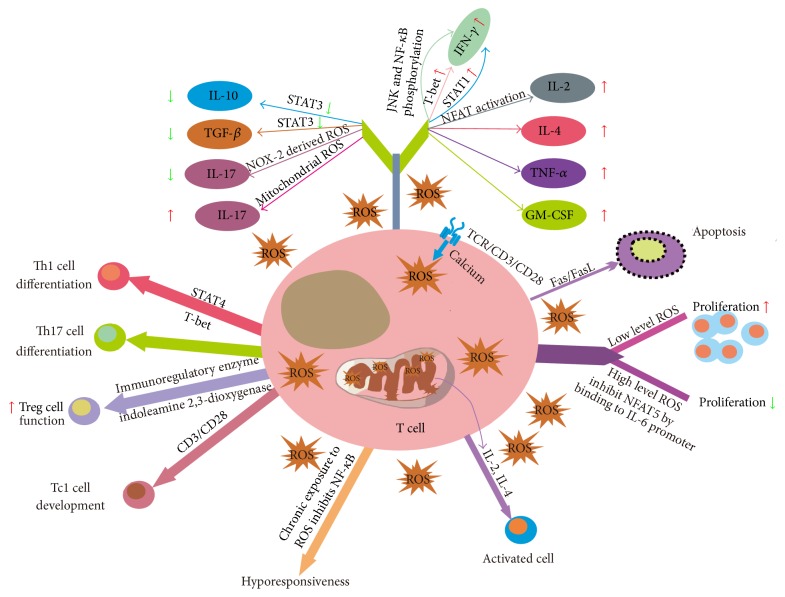
Multifaceted regulation of T cell responses by ROS. CD3 activation leads to rapid influx of calcium promoting ROS production. However, the connection between calcium and ROS production is under debate. Both signals are essential for TCR signaling. ROS trigger activation-induced cell death of T cells via Fas/FasL pathway. The low levels of mitochondrial ROS are required for T cell proliferation, while high levels of ROS inhibit NFAT5 by binding to IL-6 promoter and decrease T cell proliferation. Mitochondrial ROS are indispensable for T cell activation by regulating IL-2 and IL-4 secretion. Chronic exposure to ROS may inhibit NF-*κ*B phosphorylation and activation, which induces T lymphocytes hyporesponsiveness. NOX-2 derived ROS increase IFN-*γ* production via increasing the levels of JNK and NF-*κ*B phosphorylation, transcription factors STAT-1 and T-bet, and cytokines secretion of IL-2, IL-4, TNF-*α*, and GM-CSF. Further, NOX-2 derived ROS decrease phosphorylation of STAT3 and production of IL-10, TGF-*β*, and IL-17. Mitochondrial ROS regulate differentiation of Th17 cells and Th1 cells. Low levels of ROS induce the immunoregulatory enzyme, indoleamine 2,3-dioxygenase, and enhance the function of Tregs. NOX/ROS is a key upstream component of CD3 and CD28 signaling pathways during Tc1 cell development.

ROS excessive in the tumor microenvironment reduce antitumor function and proliferation of T cells and increase T cell apoptosis. ROS produced by other cells can reach T cells and cause oxidative stress which may induce T cell hyporesponsiveness in cancer patients [83]. It has been reported that exposure of T cells to high level ROS downregulates T cell activity [84]. Though exact effect of ROS on T cells function remains unclear, the balance between production and consumption of ROS is an important factor that determines the T cell apoptosis, activation, differentiation, proliferation, and function (Figure 2). Indeed, ROS at a low-concentration are essential for T cell activation, expansion, and effector function [34, 44].

TCR signaling pathways are affected differentially by physiological levels of ROS that trigger several proximal and distal signaling pathways in T cells. CD3 activation leads to rapid influx of calcium, in turn regulating ROS production [49], while Devadas shows that calcium release is essential for ROS production [34]. However, both signals are essential for T cell receptor signaling [85]. MnSOD/SOD2 participates in downregulation of TCR-induced prooxidative intracellular status. Several studies demonstrate that MnSOD regulates T cell differentiation and function through reducing activation-induced ROS production [82, 86]. Mitochondrial ROS control T cell activation by regulating IL-2 and IL-4 expression, which are determined by an oxidative signal originating from mitochondrial respiratory complex I [87]. In addition, complex I of the mitochondrial ETC is the source of activation-induced ROS formation [43]. However, mitochondrial ROS specifically derived from complex III are required for CD4^+^ T cell activation and antigen specific T cells expansion [49]. NOX-2-deficient T cells showed enhanced Erk kinase activation and T helper type I cytokine secretion [44]. Moreover, recently it has been shown that retrograde electron flow and ROS production were important not only in T cell activation but also in aging and development of Parkinson disease [88, 89]. In the beginning of the 90th, it has been shown that ROS could activate NF-*κ*B, while chronic exposure to ROS would inhibit NF-*κ*B phosphorylation and activation in T cells [90, 91]. In the cytoplasm, an oxidative environment for NF-*κ*B nuclear translocation is needed and in the nucleus a reducing environment for NF-*κ*B DNA binding is required. Therefore, induction of low ROS levels rather enhances NF-*κ*B (the cytosol becomes oxidizing the nucleus that is still reducing) whereas high ROS levels inhibit NF-*κ*B (cytosol and nucleus are oxidizing) [92, 93]. Reduced ROS production in association with decreased levels of JNK and NF-*κ*B phosphorylation has an impact on both IFN-*γ* and CD39 expression of CD8^+^ T cells [94]. Reduced ROS production by antioxidants or NOX inhibitors also induced Treg hypoactivation* in vitro* [95]. Tregs can suppress cysteine release from DCs, leading to oxidation of surface thiols, thereby decreasing intracellular GSH and DNA synthesis in conventional T cells. High levels of ROS could also inhibit mTOR pathway that is crucial in T cell activation and metabolism [96]. In addition, ROS can regulate proline-rich tyrosine kinase 2 (Pyk2) phosphorylation in cytotoxic T lymphocytes (CTL) by Ca^2+^-dependent pathways and Erk signaling [97]. For another, Granzyme B secreted by cytotoxic T cells induces proapoptotic pathways and then leads to cell death [98]. ROS produced by extramitochondria are involved in the process of Granzyme B induced cell death, most probably through activation of NOX [99]. Glutathione peroxidase 4 (Gpx4) could function as a unique antioxidant enzyme to inhibit lipid peroxidation and play a vital role in the homeostatic survival of CD8^+^ T cells and in both CD4^+^ and CD8^+^ T cell expansion upon TCR triggering in response to infection by preventing membrane lipid peroxidation and ferroptosis [100].

## 5. ROS Affect T Cell Differentiation

To explore effects of ROS on T cells differentiation, T cells with specific NOX-2 knockout or other ROS producing enzymes knockout have been studied. NOX-2 is composed of gP91^phox^ and p47^phox^, so mice lacking either component have been identified as good models to study the role of NOX-2 derived ROS in T cells differentiation. p47^phox^ deficiency in T cells diminished the expression of transcription factors STAT1, STAT4, and T-bet and reduced the production of cytokine, such as IL-2, IL-4, IFN-*γ*, TNF-*α*, and GM-CSF [101]. In contrast, increased phosphorylation of STAT3 and production of IL-10, TGF-*β*, and IL-17 were further observed in p47^phox^ deficient T cells [101]. Surprisingly, CD4^+^ T cells from gP91^phox−/−^ mice displayed Th1 phenotype [102]. However, both studies have detected decreased IL-4 and increased IL-17 production in NOX-2-deficient cells, suggesting a possible role of NOX complex in Th17 cell differentiation. Indeed, specific mitochondria ROS inhibitors such as N-acetylcysteine and mitoquinone reduced production of Th17 cells [103], whereas mitochondrial ROS were historically thought to be primarily cytotoxic by directly damaging DNA, lipids, and proteins [104]. Moreover, gene IEX-1 deficiency facilitated Th17 cell differentiation during early responses, which was mediated by increased formation of ROS at mitochondria following T cell activation [103].

## 6. ROS Affect T cell Apoptosis

Mitochondrial ROS are indispensable for T cell activation-induced expression of Fas ligand (FasL) that mediates activation-induced cell death (AICD) [43, 82]. Different sources of ROS are involved in AICD of T cells. TCR-stimulated upregulation of FasL and subsequent AICD was dependent upon superoxide anion, but independent of H_2_O_2_ [34]. ROS induce the expression of FasL that further activates NOX-2, which participates in the apoptotic program via ROS-mediated AKT activation and MEK inhibition [105]. Programmed death-1 (PD-1) is described initially as a marker of apoptosis and is considered as a checkpoint that controls T cell function. PD-1 blockade has been recently approved to treat patients with advanced-stage cancers by enhancing antitumor T cell immunity [106]. As the expression level of PD-1 is correlated with production of cellular ROS and oxidative metabolism [107], it would be interesting to explore potential strategies of combining ROS scavenger with PD-1 signaling blockade for rapid clinical translation.

The susceptibility of human T cells to H_2_O_2_-induced apoptosis strongly varies among T cell subsets. T cells resistance to exogenous H_2_O_2_ decreases in the following order: effector T cells > regulatory T cells > naive T cells > memory T cells [108]. CD8^+^ effector memory T cells are more sensitive to ROS compared with other T cells types [109]. It is likely that effector T cells are most insensitive to ROS-mediated death. Several studies have shown that GSH plays essential roles in increasing T cell function and proliferation [15, 110], while ROS scavenger could reduce ROS-induced apoptosis of naive and memory cells. Furthermore, a correlation between intracellular GSH depletion and progression of apoptosis has been confirmed in several studies [111–113]. Additionally, high GSH levels are associated with an apoptotic resistant phenotype in different cells. In general, TCR-stimulated ROS generation in T cells serves to regulate a proapoptotic pathway (FasL-mediated) and a proliferative pathway (ERK-mediated) that are critical for T cell function and survival.

Given importance of nuclear factor of activated T cell 5 (NFAT5) in T cell proliferation and survival [114], inhibition of binding of NFAT5 to IL-6 promoter by ROS may participate in the regulation of T cell responses. In addition, oxidative stress is a central regulator of HMGB1 translocation, release, and activity [115]. For example, mitochondrial ROS oxidation releases high mobility group box 1 (HMGB1) during apoptosis, while both intracellular and extracellular HMGB1 play pivotal roles in regulating T cell immune responses [116].

## 7. Conclusions and Perspectives

ROS produced mainly by tumor cells and immunosuppressive cells in the tumor microenvironment may determine the activation, proliferation, differentiation, and apoptosis of antitumor T cells. Considering the ROS-mediated immunosuppressive mechanisms, an important implication of therapeutic strategy targeting ROS is using antioxidant agents or supplements which may regulate antitumor T cell responses. Specifically, T cell-based therapy combined with ROS scavenger would improve clinical efficacy by enhancing expansion and function of antitumor T cells. Despite remarkable progress in recent years, the mechanism for the roles of ROS in T cell biology still remains unclear. Development of more effective strategies combining ROS manipulation and T cell-based therapy warrants further investigations particularly for the treatment of patients with advanced cancer.
